# Exhaled propofol monitoring for plasma drug prediction in rats

**DOI:** 10.3389/fvets.2025.1540413

**Published:** 2025-02-12

**Authors:** Xiaoxiao Li, Pan Chang, Xing Liu, Yi Kang, Zhongjun Zhao, Yixiang Duan, Wensheng Zhang

**Affiliations:** ^1^Department of Anesthesiology, West China Hospital, Sichuan University, Chengdu, China; ^2^Laboratory of Anesthesia and Critical Care Medicine, National-Local Joint Engineering Research Centre of Translational Medicine of Anesthesiology, West China Hospital, Sichuan University, Chengdu, China; ^3^School of Mechanical Engineering, Sichuan University, Chengdu, China

**Keywords:** propofol, rat, exhaled breath, pharmacokinetics, VUV-TOF MS

## Abstract

While propofol can be detected in exhaled breath in rats, robust evidence supporting its correlation with plasma concentrations or its use in predicting plasma levels remains lacking. In this study, eighteen mechanically ventilated rats were divided into three groups and injected with low (Group BL, *n* = 6), medium (Group BM, *n* = 6), or high (Group BH, *n* = 6) doses of propofol. The propofol concentration in exhaled breath (Ce-pro) was determined online using vacuum ultraviolet time-of-flight mass spectrometry (VUV-TOF MS), while the propofol concentration in plasma (Cp-pro) were measured using high-performance liquid chromatograph. The results indicated that after propofol injection, the peak Ce-pro was 5.87 ± 1.67 ppbv, 16.54 ± 7.22 ppbv, and 25.40 ± 3.68 ppbv, respectively. Across the different dose groups, C_max_ of Ce-pro and Cp-pro were linearly correlated (*P*_BL_ = 0.032, *P*_BM_ = 0.031, *P*_BH_ = 0.049). T_max_ of Ce-pro was 1.22 ± 0.17 min, 1.28 ± 0.13 min, and 1.33 ± 0.01 min, respectively (*P* = 0.341), similar to the T_max_ of Cp-pro (1.00 ± 0.00 min). After natural logarithm transformation, the correlation between LN(Ce-pro) and LN(Cp-pro) was well fitted by a linear model, with $R_{BL}^{2}$ = 0.94, $R_{BM}^{2}$ = 0.95, $R_{BH}^{2}$ = 0.98, and $R_{ALL}^{2}$ = 0.96. Using the obtained regression equation LN(Cp-pro) = 1.42^*^LN(Ce-pro)-1.70, the predicted Cp-pro values showed excellent agreement with the actual values within groups (ICC_BL_ = 0.92; ICC_BM_ = 0.97, ICC_BH_ = 0.99, all *P* < 0.001). This study demonstrates a strong correlation between exhaled and plasma propofol concentrations in rats, indicating that exhaled concentrations can be effectively used to estimate plasma levels.

## Introduction

Propofol, a widely used intravenous anesthetic in animal anesthesia, is favored for its rapid onset and effective anesthetic properties (1, 2). Frequent blood sampling during pharmacokinetic (PK) analysis in rats poses significant challenges for operators, requiring a high level of technical proficiency. Improper techniques can lead to blood contamination or sampling failure, particularly in small animals. Additionally, due to limitations in analytical methods, monitoring propofol concentrations in plasma (Cp-pro) online remains challenging. Previous studies have shown that propofol can be detected in exhaled breath. Instruments such as selected ion flow tube mass spectrometry (SIFT-MS) (3), ion molecule reaction mass spectrometry (IMR-MS) (4), ion mobility spectrometry (IMS) (5), and gas chromatography combined with surface acoustic sensor (GC-SAW) (6) have been explored for exhaled propofol concentration (Ce-pro) monitoring. However, these instruments are often characterized by their large size and high noise levels. Furthermore, robust evidence supporting its correlation with plasma concentrations or its use in predicting plasma levels remains lacking.

Our team developed a mobile exhaled propofol concentration monitor utilizing vacuum ultraviolet (VUV) technology combined with time-of-flight mass spectrometry (TOF MS) (7, 8). This instrument has demonstrated its capability for online monitoring of propofol in rats, beagles, and humans. In this study, rats were administered single intravenous injections of different doses of propofol. The Ce-pro was monitored online using VUV-TOF MS, while the Cp-pro were measured using high-performance liquid chromatograph (HPLC). The aim of this study was to evaluate the consistency between Ce-pro and Cp-pro, and to predict the Cp-pro based on monitored Ce-pro values, providing foundational animal data to support future clinical studies.

## Materials and methods

### Ethics approval

Eighteen male Sprague-Dawley rats (aged 12–15 weeks and weighing 350 ± 50 g) obtained from Chengdu DOSSY Experimental Animals Co., Ltd were included in this experiment. All rats were housed in the SPF animal facility at Tianfu Life Science Park, West China Hospital, Sichuan University, with free access to food and water. The experiments began after a one-week acclimatization period. This study was approved by the Animal Ethics Committee of West China Hospital, Sichuan University (20220420001), and all procedures complied with animal welfare guidelines.

### Rat ventilation model

All rats fasted for 12 h before the experiment, with unrestricted access to water. Anesthesia was induced via intraperitoneal injection of 10% Nembutal (pentobarbital sodium, Think-Far Technology, Beijing, China) at 35 mg/kg. Once the righting reflex disappeared, the rats were positioned supine on a heated plate, and their tail veins were punctured and catheterized. A tracheotomy was performed for mechanical ventilation (R407, RWD Life Technology, Shenzhen, China), with the trachea and tracheal catheter firmly secured to prevent air leakage. Ventilator parameters included pure oxygen supply, a respiratory rate of 60 breaths/min, a tidal volume of 1.5 mL/100 g body weight, and an inhalation-to-exhalation ratio of 1:1. One femoral artery was catheterized for blood pressure monitoring (BL-420 F, Chengdu Techman, Chengdu, China), and another for blood sample collection. The rats' rectal temperature was measured using a probe (BeneView T8, Shenzhen Mindray, Shenzhen, China), and body temperature was kept at 36.5 ± 0.5°C. The heating plate temperature was adjusted if the temperature of rats exceeded 37°C or fell below 36°C. Pentobarbital sodium was re-administered intraperitoneally at one-third of the initial dose every 30 min to maintain anesthesia. Additionally, 0.9 % saline was continuously infused at a rate of 8 mL/kg/h.

### Administration method and dosage

The sample size was determined based on the “Guiding Principles for Registration Review of Animal Testing in Medical Device Studies” issued in China (9), along with the requirements of pharmacological experiments. Accordingly, each group consisted of six rats. A total of eighteen rats were randomly divided into three groups using a random number table: low-dose (Group BL, *n* = 6), medium-dose (Group BM, *n* = 6), and high-dose (Group BH, *n* = 6) propofol bolus groups. The propofol doses were 1 ED_50_ (6 mg/kg, the dose required to abolish the righting reflex in 50% of the rats) for Group BL, 2 ED_50_ (12 mg/kg) for Group BM, and 4 ED_50_ (24 mg/kg) for Group BH (10). Propofol across all three groups was administered at a constant rate of 0.4 mL per 30 s using a microinfusion pump (R462, RWD Life Science, Shenzhen, China).

### Ce-pro monitoring

**Figure 1:**
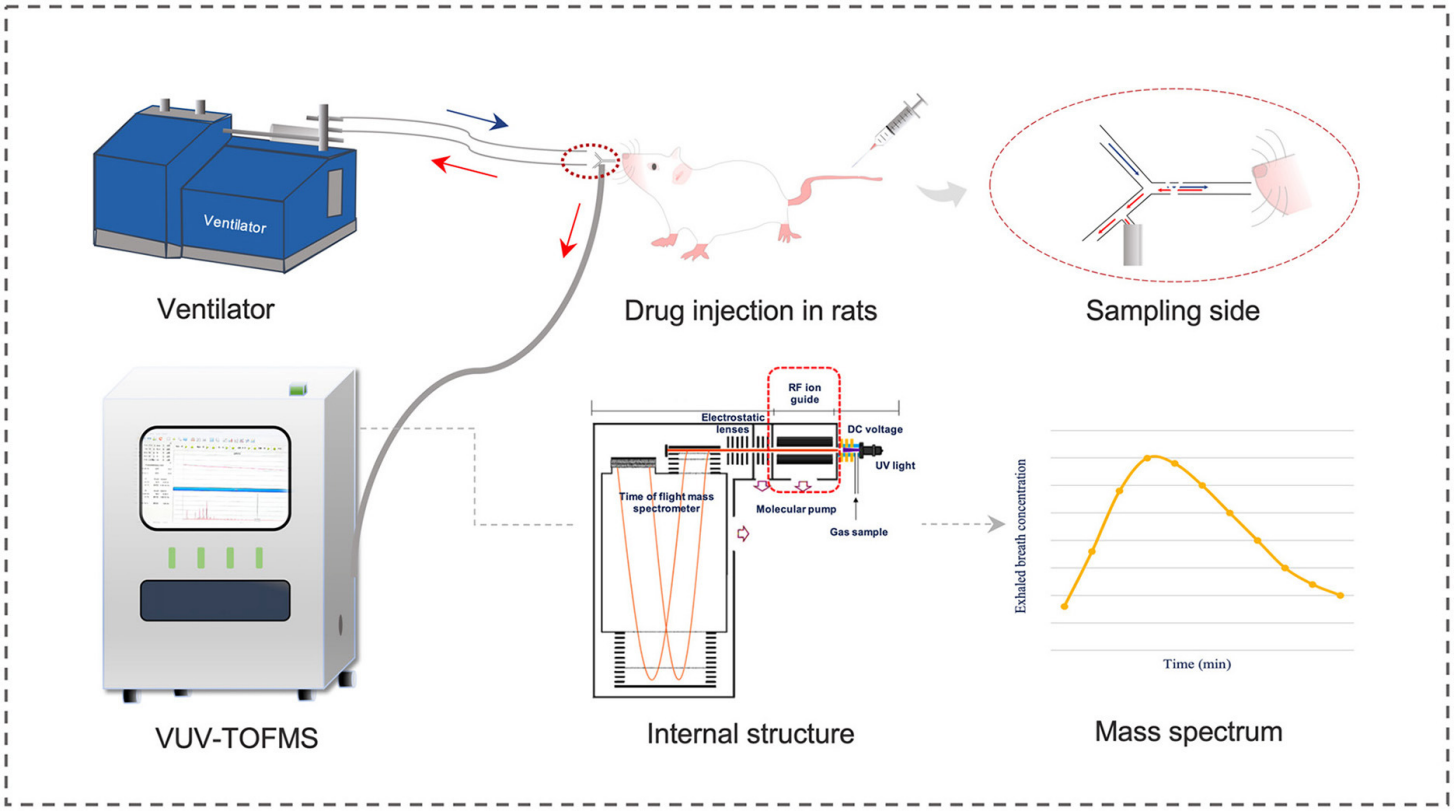
Schematic diagram of VUV-TOF MS for monitoring the exhaled propofol concentration in rats.

The structure, operating principles, and calibration method of the VUV-TOF MS system have been detailed in our previous study (11). As shown in Figure 1, a polyurethane tracheal catheter was connected to the VUV-TOF MS instrument via a polycarbonate three-way valve within a respiratory circuit made of polytetrafluoroethylene. Detection conditions were set as previously described, enabling continuous monitoring of propofol at 20-s intervals from the start of administration to 120 min post-administration. An m/z value of 177.6 was identified, and the concentration of propofol in exhaled air was calculated using the calibration curve.

### Cp-pro monitoring

During the experiment, 0.2 mL of femoral artery blood was collected into EP tubes at specific time points: before propofol administration and at 1, 3, 5, 10, 20, 30, 60, 90, and 120 min post-administration. After each sampling, an equal volume of stored blood (0.2 mL) was reinjected intravenously via the tail vein. Once all samples from a rat were collected, the blood samples were centrifuged at 3,500 rpm for 10 min at 4°C. The supernatant (100 μL) was then extracted and stored at −80°C. The Cp-pro was determined using a HPLC system (Waters, Waters Corporation, USA).

The chromatographic conditions were as follows: a Swell Chromplus C18 column (150 mm × 4.6 mm, 5 μm) was used, with the column temperature maintained at 30°C. The mobile phase consisted of pure water and acetonitrile mixed in a 38:62 (v/v) ratio. Fluorescence detection was performed at an excitation wavelength of 276 nm and an emission wavelength of 310 nm. The flow rate was kept constant at 1.0 mL/min, and the injection volume was 10 μL. Retention times were determined to be 3.9 min for thymol (used as the internal standard) and 7.4 min for propofol.

### Statistical analysis

Statistical analyses were conducted using GraphPad Prism (Version 9.4, GraphPad Software, USA). Data are expressed as mean ± standard deviation or median (interquartile range). One-way analysis of variance was used for comparisons among three groups. Spearman correlation analysis was performed to evaluate the relationship between Ce-pro and Cp-pro, with the correlation coefficient (*r*) calculated. Furthermore, the natural logarithms (LN) of Ce-pro and Cp-pro were calculated, followed by a linear correlation analysis to determine the *R*^2^. The intraclass correlation coefficient (ICC) was calculated to assess the correlation of predicted Cp-pro based on the regression model from all groups and the actual Cp-pro values. The PK model and parameters were calculated using Phoenix WinNonlin software (Phoenix WinNonlin, Certara, USA). Concentrations below the limit of detection (LOD) were treated as 0 before reaching C_max_ and as not detectable after reaching C_max_. A two-step approach was employed to estimate the PK parameters. Individual data were fitted to one-compartment, two-compartment, and three-compartment models. Model parameters were estimated using the least-squares method. Comparisons between Ce-pro and Cp-pro for T_1/2_, T_1/2_, K_10_, K_12_, and K_21_ were performed using the *t*-test. However, no comparisons were made for V_1_, CL, and AUC_0 − 120_. A *P* < 0.05 was considered statistically significant.

## Results

A total of 18 male rats from the same batch were included in this experiment, with mean body weights of 344 ± 21 g in Group BL, 330 ± 32 g in Group BM, and 318 ± 19 g in Group BH (*P* = 0.218). The administered doses were 2.07 ± 0.13 mg in Group BL, 4.76 ± 0.39 mg in Group BM, and 7.63 ± 0.46 mg in Group BH.

### Validation of the determination method of Ce-pro

**Figure 2:**
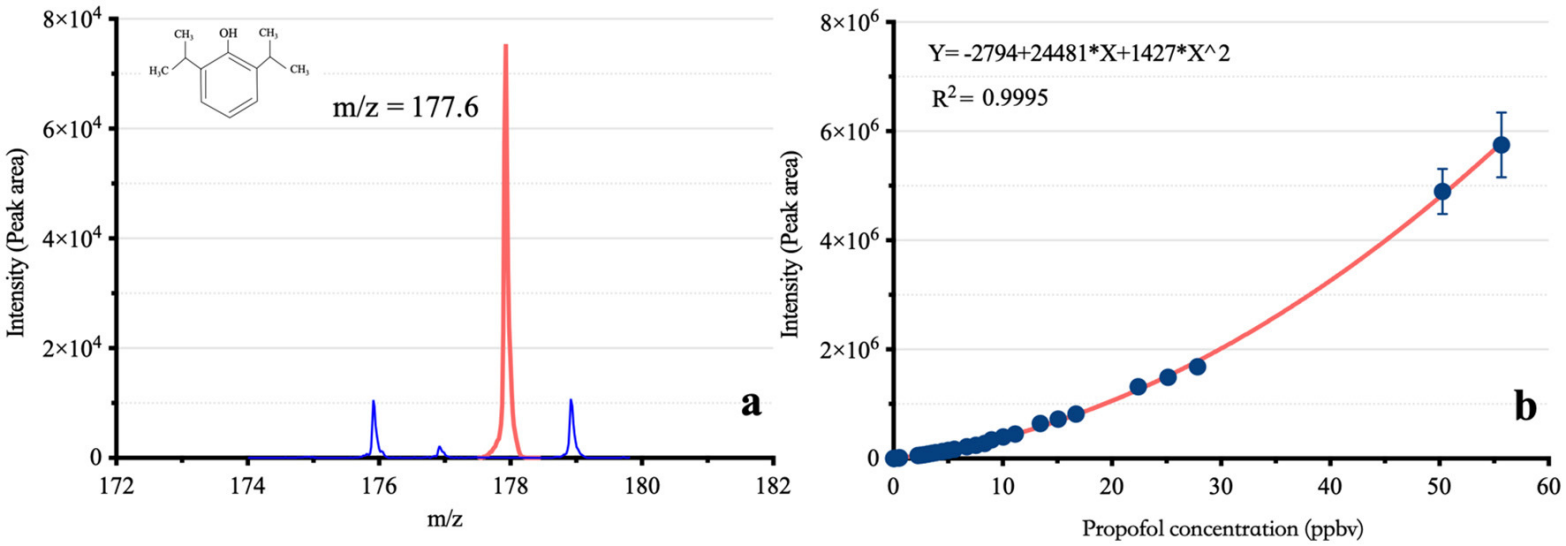
The mass-to-charge ratio and calibration curve of propofol based on VUV-TOF MS. m/z: Mass-to-charge ratio; **(A)** shows the m/z of propofol in the VUV-TOF MS, and **(B)** presents its calibration curve, following a second-order polynomial distribution.

As shown in Figure 2, the m/z of propofol detected by the VUV-TOF MS was 177.6. For measured concentrations ranging from 0.04 ppbv to 55.68 ppbv, the correlation between signal intensity and concentration followed a second-order polynomial distribution. The calibration curve was fitted to the equation y = −2,794 + 24,481^*^x + 1,427^*^x^2^ (*R*^2^ = 0.9995). The LOD was 0.04 ppbv, and the limit of quantification (LOQ) was 0.12 ppbv, respectively.

### The PK models and parameters of Ce-pro and Cp-pro

**Figure 3:**
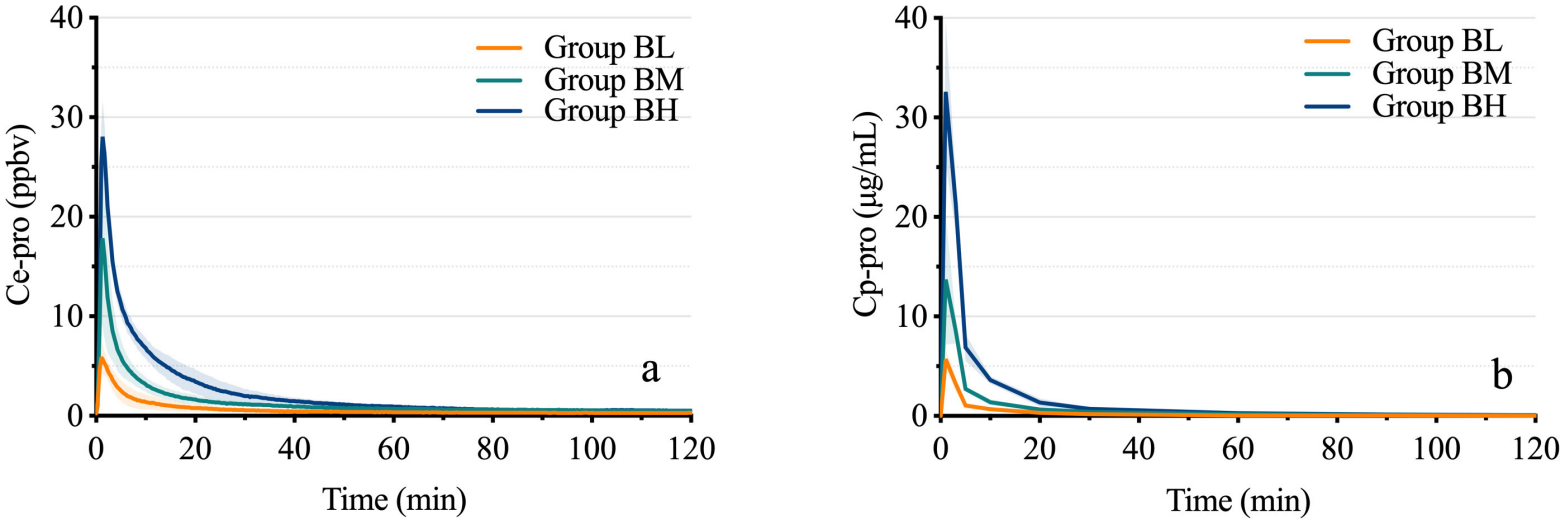
Time-concentration curves of propofol in exhaled breath and plasma. **(A)** displays the time-concentration curves of propofol in exhaled breath at different doses, while **(B)** shows the corresponding curves in plasma. Ce-pro, Propofol concentration in exhaled breath; ppbv, Parts per billion by volume; Cp-pro, Propofol concentration in plasma; Group BL, Low-dose bolus injection; Group BM, Medium-dose bolus injection; Group BH, High-dose bolus injection.

As shown in Figure 3, the average C_max_ of Ce-pro in the three groups was 5.87 ± 1.67 ppbv, 16.54 ± 7.22 ppbv, and 25.40 ± 3.68 ppbv, respectively, while the average C_max_ of Cp-pro was 5.71 ± 0.94 μg/mL, 13.71 ± 6.53 μg/mL, and 32.59 ± 7.17 μg/mL, respectively. Across the different dose groups, the C_max_ of Ce-pro and Cp-pro showed a linear correlation (*P*_BL_ = 0.032, *P*_BM_ = 0.031, *P*_BH_ = 0.049). The T_max_ of Ce-pro was 1.22 ± 0.17 min, 1.28 ± 0.13 min, and 1.33 ± 0.01 min, respectively (*P* = 0.341), which was similar to the T_max_ of Cp-pro (1.00 ± 0.00 min). As shown in Table 1, the Ce-pro and Cp-pro data were well described by the two-compartment model. No significant differences were observed between the Ce-pro and Cp-pro groups for the T_1/2α_, K_12_, and K_21_ parameters. However, the K_10_ values in Ce-pro were significantly lower than those in Cp-pro (*P*_BL_ < 0.001, *P*_BM_ = 0.045, *P*_BH_ = 0.006).

**Table 1:** The PK parameters of Ce-pro and Cp-pro.

	**Group BL (*****n*** = **6)**			**Group BM (*****n*** = **6)**			**Group BH (*****n*** = **6)**		
**Parameter**	**Ce-pro**	**Cp-pro**	***P***	**Ce-pro**	**Cp-pro**	***P***	**Ce-pro**	**Cp-pro**	***P***
V_1_, mg/(conc_unit)	0.29 (0.09)	0.25 (0.05)	/	0.22 (0.12)	0.21 (0.10)	/	0.24 (0.05)	0.19 (0.04)	/
CL, mg/(min^*^conc_unit)	0.02 (0.01)	0.06 (0.01)	/	0.02 (0.01)	0.04 (0.01)	/	0.03 (0.01)	0.03 (0.01)	/
AUC_0 − 120_, min^*^conc_unit	108.14 (30.44)	38.08 (6.92)	/	194.48 (34.72)	95.19 (12.91)	/	289.10 (37.97)	229.27 (42.13)	/
T_1/2α_, min	3.06 (1.31)	1.81 (0.49)	0.058	2.31 (0.70)	2.11 (0.83)	0.685	2.54 (1.06)	2.04 (0.41)	0.307
T_1/2β_, min	74.92 (29.66)	29.57 (10.96)	0.007	40.67 (10.88)	46.13 (37.51)	0.739	25.83 (12.74)	34.66 (23.15)	0.432
K_10_, 1/min	0.08 (0.04)	0.23 (0.05)	< 0.001	0.12 (0.05)	0.25 (0.14)	0.045	0.11 (0.02)	0.19 (0.05)	0.006
K_12_, 1/min	0.16 (0.06)	0.16 (0.05)	0.946	0.18 (0.07)	0.13 (0.07)	0.312	0.14 (0.07)	0.12 (0.04)	0.599
K_21_, 1/min	0.04 (0.01)	0.05 (0.02)	0.252	0.05 (0.01)	0.03 (0.02)	0.084	0.10 (0.07)	0.08 (0.08)	0.542

Data are expressed as the mean (SD). Group BL, bolus injection of low dose; Group BM, bolus injection of medium dose; Group BH, bolus injection of high dose. Ce-pro: propofol concentration in exhaled breath; Cp-pro, propofol concentration in plasma; “conc_unit” in Ce-pro represented ppbv, while in Cp-pro represented μg/mL; V_1_, the apparent volume of the central compartment; CL, total body clearance; AUC_0 − 120_, area under the curve of the propofol concentration in exhaled breath or blood vs. time; T_1/2α_, the half-life of distribution; T_1/2β_, the half-life of elimination; K_10_, the rate constant describing elimination from the central compartment to outside; K_12_, the rate constant describing distribution from the central compartment into peripheral compartment; K_21_, the rate constant describing redistribution from the peripheral compartment into the central compartment. For parameters T_1/2α_, T_1/2β_, K_10_, K_12_ and K_21_, comparisons between C_E_ and C_p_ were conducted with a *t* test. For the V_1_, CL, and AUC_0 − 120_ parameters, no comparisons were performed.

### Correlation between the Ce-pro and Cp-pro

**Figure 4:**
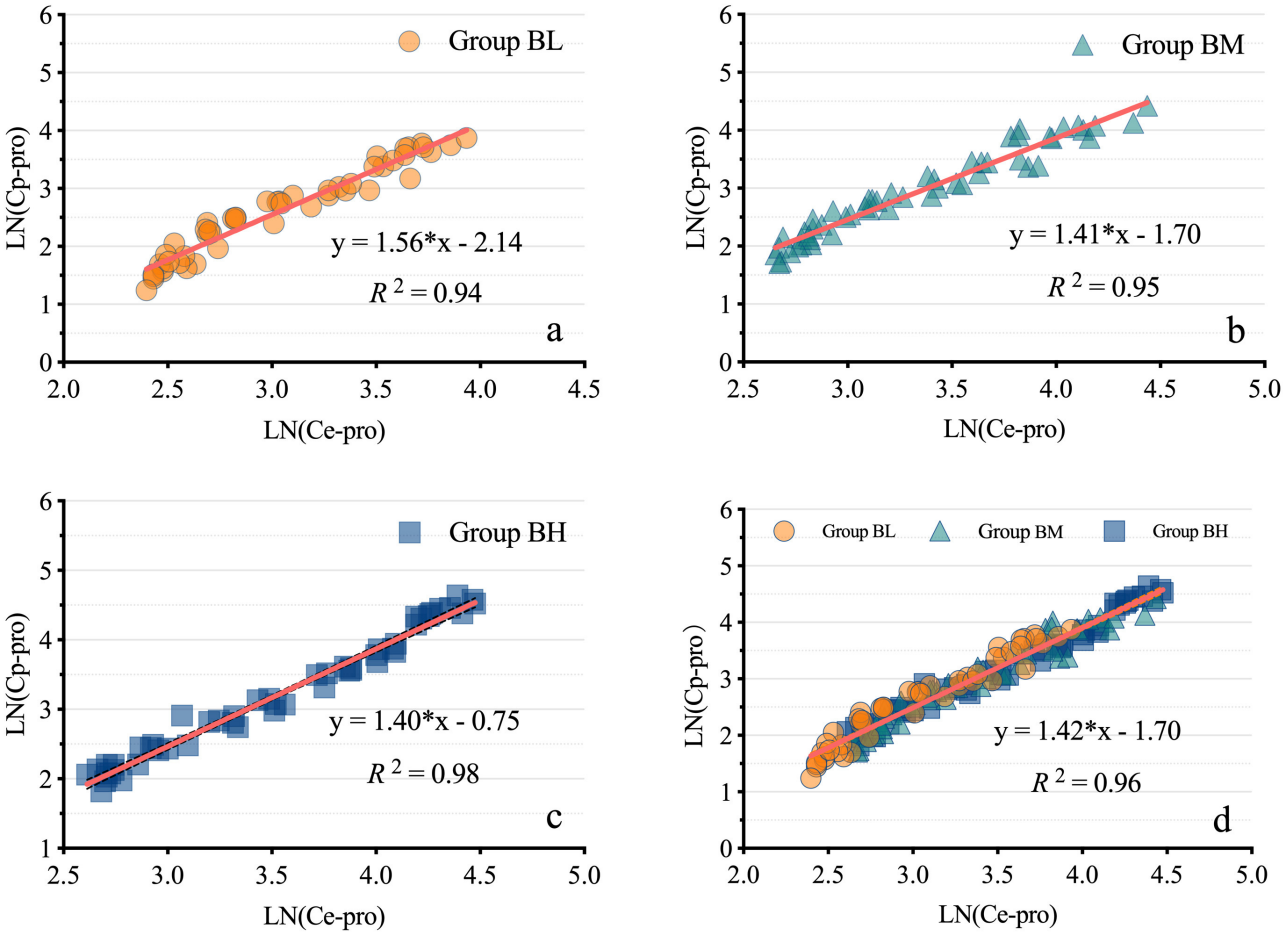
Linear regression models of propofol in exhaled breath and plasma. **(A–D)** display the linear regression models of propofol in exhaled breath and plasma for low, medium, high, and combined dosages, respectively. LN(Ce-pro), Natural logarithm of propofol concentration in exhaled breath; LN(Cp-pro), Natural logarithm of propofol concentration in plasma; Group BL, Low-dose bolus injection; Group BM, Medium-dose bolus injection; Group BH, High-dose bolus injection.

**Figure 5:**
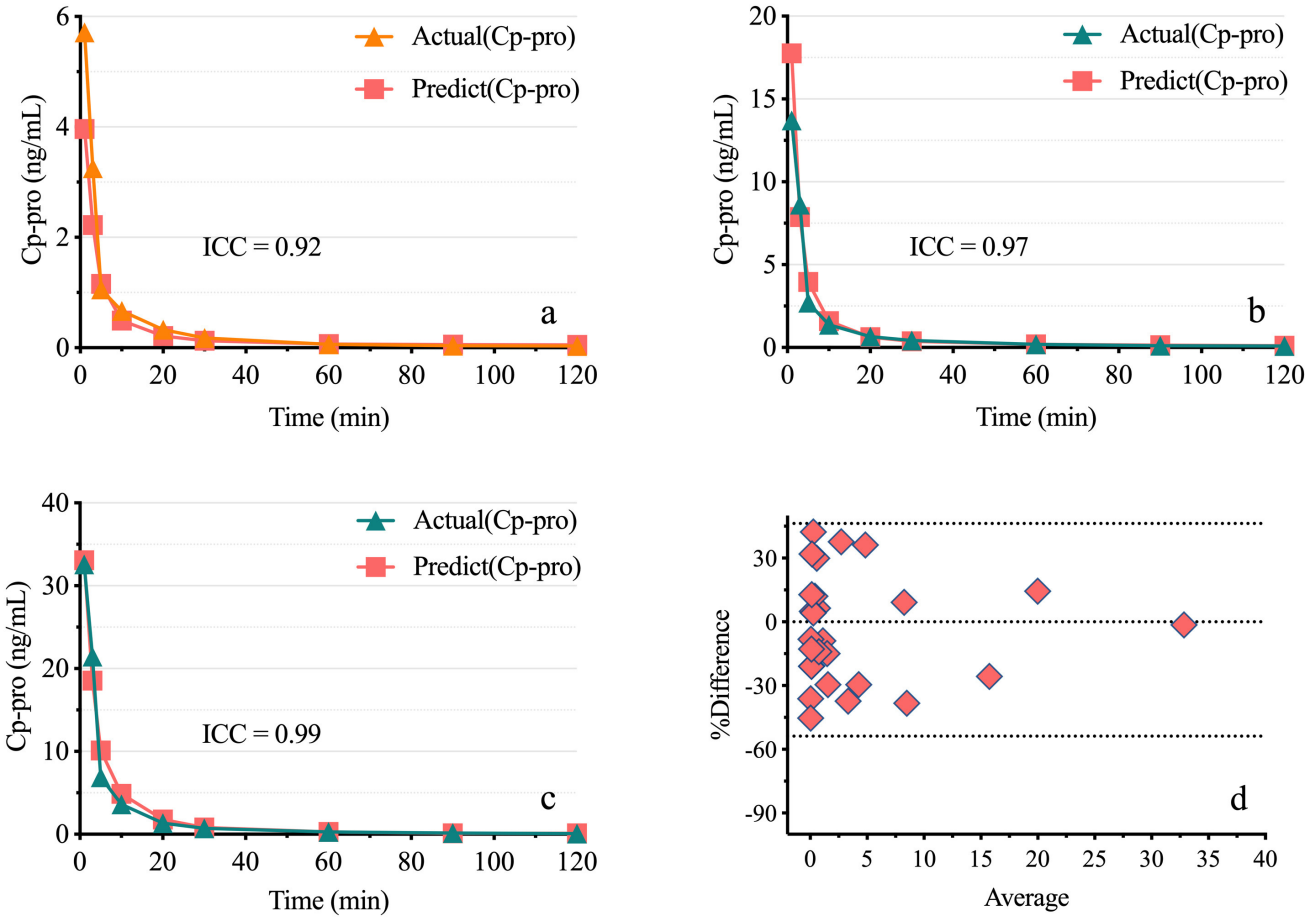
Consistency of predicted plasma propofol levels Based on exhaled breath measurements. **(A–C)** show the actual and predicted plasma propofol concentrations for low, medium, and high doses. Actual (Cp-pro), Measured plasma concentration; Predict (Cp-pro), Predicted plasma concentration. **(D)** presents the Bland-Altman plot, with points evenly distributed around the midline, indicating good consistency. Group BL, Low-dose bolus injection; Group BM, Medium-dose bolus injection; Group BH, High-dose bolus injection.

The results demonstrated a strong correlation between Ce-pro and Cp-pro, with Spearman *r*_BL_ = 0.98, *r*_BM_ = 0.98, *r*_BH_ = 0.99, and *r*_ALL_ = 0.98, all *P* < 0.001. As illustrated in Figure 4, after applying the LN transformation to Ce-pro (pptv) and Cp-pro (ng/mL) values, the linear relationship between LN(Ce-pro) and LN(Cp-pro) was well described, with $R_{\text{BL}}^{2}$ = 0.94, $R_{\text{BM}}^{2}$ = 0.95, $R_{\text{BH}}^{2}$ = 0.98, and $R_{\text{ALL}}^{2}$ = 0.96, all *P* < 0.001. Furthermore, based on the obtained overall regression equation LN(Cp-pro) = 1.42^*^LN(Ce-pro)-1.70, the monitored Ce-pro were used to predict Cp-pro. As illustrated in Figure 5, the intraclass correlation coefficients (ICC) indicated excellent agreement between predicted and actual values within groups (ICC_BL_ = 0.92; ICC_BM_ = 0.97, ICC_BH_ = 0.99, all *P* < 0.001.)

## Discussion

This experiment successfully detected the exhaled propofol concentration of rats in real time using the VUV-TOF MS system and obtained estimated PK parameters. Furthermore, a strong correlation was established between exhaled and plasma propofol concentrations, providing a technical basis and animal data for online monitoring of the propofol concentration in future clinical studies.

Individualized dose adjustment based on therapeutic drug monitoring is a critical foundation of precision medicine (12). The MAC of inhalational anesthetics can be measured online using infrared sensors that detect their specific absorption wavelengths (13, 14). However, no suitable instruments are currently available for monitoring the concentration of intravenous anesthetics. The traditional GC-MS method is time consuming, which makes it impossible to achieve real-time detection to meet the needs of clinical anesthesia or point-of-care testing (15). The bispectral index, which reflects the cerebral effects of propofol, can also be unreliable because it can be easily affected by multiple factors, such as the application of an external cardiac pacemaker (16), changes in body position (17, 18), or the combination of other drugs, such as ketamine (19). In 2003, Harrison and his colleagues first discovered that propofol can be measured in exhaled breath by proton transfer reaction-mass spectrometry (PTR-MS) at the ppbv level (20). This finding led anesthesiologists to recognize propofol as a semivolatile organic compound, prompting studies on its concentration in exhaled breath (21). However, compared with sevoflurane (156.9 mmHg at 20°C) or water (17.5 mmHg at 20°C), propofol exhibits relatively low volatility due to its low saturated vapor pressure (0.142 mmHg at 20°C) (22–24). Our team developed a compact MS with high sensitivity of up to pptv level. Compared with the GC-MS analytical method, this system does not require preseparation of air samples, and it achieves a signal response within 1 s. In our study, a measurement period of 20 s was used to avoid intensity errors caused by sudden ventilation changes, thereby improving the stability of the output results. Compared to other online analytical instruments, such as PTR-MS and SIFT-MS, this VUV-TOF-MS instrument features a straightforward and intuitive operation, making it easier for anesthesiologists to use. Although the calibration curve of signal intensity vs. concentration (0.04 ppbv−55.68 ppbv) is nonlinear, it follows a second-order polynomial distribution with a high fitting degree (*R*^2^ = 0.9995). Additionally, the calibration results were robust, with intraday variability < 15 %, meeting the requirements of sample analysis. Berchtold et al. emphasized that sensitivity, selectivity, scan speed, and robustness are critical factors for real-time monitoring of exhaled drugs, all of which are satisfactorily fulfilled by the method of VUV-TOFMS detection (25). The nonlinear standard curve is attributed to the saturated adsorption of propofol molecules on the instrument pipeline. The proportion of adsorption is high at low concentrations but decreases at high concentrations, resulting in an upward-opening calibration curve (26).

Compared with other reported results, the correlations between Ce-pro and Cp-pro range from moderate to strong (*R*^2^ = 0.58–0.98) (6, 27–29), in our study, the correlations were significantly stronger. One possible explanation is the use of a highly sensitive analytical instrument, the VUV-TOF MS, which has a much lower LOD of 0.04 ppbv compared to Multi-Capillary Column Ion Mobility Spectrometry (MCC-IMS), which has an LOD of 0.1 ppbv (30). Another factor is the use of a heated polyether ether ketone (PEEK) tube (outer diameter: 2.5 mm, inner diameter: 2 mm, length: 150 mm), which was insulated with thermal foam and connected to a temperature control switch to maintain a constant temperature of 100°C. This tube delivered gas samples under negative pressure into the ionization chamber. The PEEK material's high melting point, excellent thermal stability, and strong chemical resistance minimized the adsorption of propofol, thereby preserving sample integrity. Maurer et al. investigated the adsorption and desorption behaviors of various tubing materials with respect to propofol, including perfluoroalkoxy (PFA), polytetrafluoroethylene (PTFE), polyurethane (PUR), silicone, and Tygon (31). Their findings identified PFA as the most suitable material for measuring the propofol concentration in exhaled breath. However, our preliminary tests demonstrated that PEEK tubing exhibited even lower propofol adsorption compared to PFA. In contrast, the unheated and relatively long PTFE sampling tubes commonly used with MCC-IMS in ventilator setups were found to increase the likelihood of propofol gas loss (32). Finally, the sampling rate also contributed to the improved results. To minimize the effects of sudden airflow changes on concentration measurements, we used a 20-s sampling interval instead of a 1-s interval. This approach provided a mean concentration value calculated about 20 measurements, significantly enhancing the accuracy compared to single-measurement analysis.

We explored the PK models and parameters of the propofol concentration in exhaled breath following bolus injections at varying doses. The low dose corresponded to the ED_50_ for the loss of the righting reflex in rats, while the medium and high doses represented 2ED_50_ and 4ED_50_ doses, respectively. The exhaled propofol concentration peaked at ~1 min after injection and then rapidly decreased, conforming to a two-compartment model. This finding is consistent with results reported in human studies (4, 6, 33). This is because the lung is one of the central compartments where propofol is distributed rapidly after injection. As propofol-laden blood flows through the alveoli, free and dissolved propofol volatilizes, forming vapor molecules that quickly cross the pulmonary respiratory membrane, facilitating distribution from blood vessels to the alveoli. Although the airway may participate in gas exchange, propofol, a lipophilic, hydrophobic drug with high solubility in blood, is generally believed to be the main site of propofol gas exchange (34, 35), where the capillary network is abundant, the absorption surface area of the alveoli is large, and the distance between the capillary and alveoli is very small, which is conducive to the exchange of gaseous propofol gas by diffusion (36). When the concentration of propofol in the alveoli is greater, the transpulmonary gradient is greater, and the driving pressure of propofol through the pulmonary respiratory membrane is greater. Moreover, propofol molecules in exhaled breath can be detected at the following measurement time point with no delay, indicating that the diffusion process was rapid. Some studies have shown that the presence of propofol in breath is detected later than that in blood. Our results suggest that the delay may be due to species differences or detection technology (37). Compared with the estimated PK parameters of the plasma propofol concentration, the K_10_ values in exhaled breath were significantly lower, possibly because of the lower elimination rate of propofol in the lung than in the plasma.

We further analyzed the correlation between propofol concentrations in exhaled breath and plasma, which previous studies have demonstrated to be linearly dependent (38). Our study also exhibited a strong correlation, with even better consistency compared to previous findings. This improvement can be attributed not only to the superior detection performance of the VUV-TOF MS instrument but also to the meticulous attention we paid during sample collection. A stable and consistent tidal volume is a prerequisite for online monitoring of exhaled propofol concentrations in rats. To achieve this, we performed tracheal intubation with mechanical ventilation to prevent the effects of respiratory depression on concentration measurements caused by excessive propofol. Additionally, the tracheal cannula was secured with ligatures, as closed cuffs are unavailable for rat endotracheal tubes, and ligation effectively reduces the risk of air leakage. Although we collected mixed expiratory breath from the rats instead of alveolar air, we took care to avoid significant positional changes during the procedure to minimize potential variations in alveolar dead space. Additionally, Oluwasola Lawal suggested that the ideal breathing sampling method for exhaled propofol concentration monitoring should be simple and suitable for individual physiology. Mixed expiratory breath is considered the simplest and most accessible type of breath sample (39). Furthermore, the primary purpose of monitoring exhaled drug concentrations is to accurately reflect plasma drug concentrations. Using the regression equation derived from exhaled and plasma drug concentrations at 1–4 times the ED_50_ dose, we successfully predicted plasma concentrations corresponding to various exhaled concentrations. The values of ICCs demonstrated excellent agreement between the predicted and measured values, further enhancing the practical significance of monitoring exhaled drug concentrations.

Our study also has some limitations. First, although we tried to explain the pharmacokinetics of the exhaled propofol concentration, the lung physiology is complex, and there are many factors, such as age, sex, tidal volume, respiratory frequency, respiratory mechanics, pulmonary diffusion and functional residual capacity, related to disease status that increase the complexity of the pharmacokinetics of exhaled propofol. Theoretically, it is speculated that the factors affecting lung ventilation and air exchange as well as the factors affecting the ratio of lung ventilation to blood flow can have a certain effect (40). Compared to the large intake required by instruments, the small tidal volume of rats is greatly affected by slight mechanical force. In addition, this study focused only on the correlation between the propofol concentration in exhaled breath and that in plasma and lacked data concerning the effects on the brain and depth of anesthesia. Future studies should focus on online propofol monitoring for the rapid prediction of anesthesia depth.

## Conclusions

The exhaled propofol concentration in rats can be monitored in real-time using the VUV-TOF MS. The strong correlation between exhaled and plasma propofol concentrations, well described by a linear model, indicates the potential to predict plasma propofol levels based on exhaled breath measurements. This study offers valuable insights and foundational data to support future clinical research.

## Data Availability

The original contributions presented in the study are included in the article/supplementary material, further inquiries can be directed to the corresponding author.
